# *Klebsiella pneumoniae* Carbapenemase (KPC)–Producing *K. pneumoniae* Contamination of an In-Room Sink in a New Bed Tower

**DOI:** 10.1017/ash.2021.143

**Published:** 2021-07-29

**Authors:** Bobby Warren, Becky Smith, Sarah Lewis, Deverick Anderson, Bechtler Addison

## Abstract

**Group Name:** Duke Center for Antimicrobial Stewardship and Infection Prevention

**Background:** Wastewater drains in hospital patient rooms have been identified as environmental reservoirs for multidrug-resistant organisms, and they have been linked to outbreaks of carbapenem-resistant Enterobacteriaceae (CRE). We studied the colonization of wastewater drains in a new hospital bed tower. **Methods:** A patient care unit in a new bed tower opened on July 18, 2020. In-room sinks were located in each hospital room opposite the patient head wall. Patients admitted to this unit underwent weekly rectal cultures to survey for carbapenemase-producing CRE. Additionally, infection preventionists performed routine surveillance of all clinical cultures for CRE. Cultures were performed from all patient room sinks in this unit monthly beginning September 14, 2020. Samples were obtained from the drain cover, handles, and top of bowl using sponges soaked in neutralizing buffer and processed using the stomacher technique. The tail-pipe was sampled using a flocked mini-tip swab soaked in neutralizing buffer; the P-trap water was sampled with sterile tubing attached to a 50-mL syringe. All samples were plated on HARDYCHROM-ESBL and KPC Colorex media and were incubated at 37°C for 24 hours. **Results:** The first identified CRE-positive patient was admitted to the new unit on December 4, 2020; urine culture obtained at the time of admission grew KPC–producing *Klebsiella pneumoniae* (KPC-KP). The sink in this patient’s room had been sampled 3 prior times (most recently on November 9, 2020) and was negative for CRE. On December 7, 2020, KPC-KP was found on the drain cover (6,750 colony-forming units, CFU) and in the sink’s P-trap (1,840 CFU) of the index patient’s room during routine sink surveillance. Additional samples from other room surfaces were taken on December 9, 2020, and KPC-KP was recovered from the computer keyboard (452 CFU) and patient bedrails (880 CFU). The patient was discharged from this room December 13, 2020, and the room underwent enhanced terminal room cleaning including UV-C light. On the next routine sink sampling on January 4, 2021, KPC-KP was recovered again in the index room sink P-trap (9,800 CFU) but at no additional sites. MLST was performed, and all isolates were ST-258. **Conclusions:** In a new bed tower with no prior evidence of CRE-positive patients, the first identified case of a CRE (KPC-KP) in a patient resulted in widespread environmental contamination of the room after only 3 days of hospitalization and contamination of the in-room sink drain that persisted after 1 month. Given the ease with which CRE colonizes wastewater drains, new strategies are needed to mitigate drain colonization and to prevent CRE transmission to subsequent patients.

**Funding:** No

**Disclosures:** None

